# Radial and translational motions of a gas bubble in a Gaussian standing wave field

**DOI:** 10.1016/j.ultsonch.2023.106712

**Published:** 2023-12-01

**Authors:** Yuchen Zang

**Affiliations:** School of Physics and Technology, Nanjing Normal University, Nanjing 210023, China

**Keywords:** Radial oscillation, Translational motion, Gas bubble, Gaussian standing wave

## Abstract

Acoustic waves with a finite beam width are widely used in acoustic manipulation and cavitation applications. In view of this, radial oscillation and translational motion of a gas bubble in a Gaussian standing wave field are studied in this work. Dynamic differential equations for the bubble are derived with the axial and transverse motions coupled with each other. A comprehensive numerical study is also carried out in the parameter space of the driving frequency, pressure amplitude, initial coordinate, off-axial distance and beam waist. The results demonstrate that the nonlinear radial oscillation can be intensified by a higher pressure amplitude and a smaller off-axial distance. Whether the driving frequency is much lower than the resonance frequency determines not only the final equilibrium position but also the direction of translational motion for the gas bubble. With the widening of the Gaussian standing wave, the radial oscillation will be weakened and the translational motion will be slowed down due to reduction of the pressure gradient regardless of the driving frequency. The results obtained in this study is of interest for an understanding of the bubble dynamics in non-plane acoustic wave fields.

## Highlights

• Coupled dynamic equations derived for a bubble in Gaussian standing waves.
• Transverse radiation force reverses sign with the variation of driving frequency.
• Axial and transverse motions weakened with the widening of wave front.

## 1 Introduction

A gas bubble subject to an ultrasonics wave field exhibits both radial oscillation and translational motion [1]. Unlike the radial oscillation driven by the temporal variation of the wave field, the translational motion is generated by the spatial gradient of the ambient acoustic pressure [2]. It is noted that the radial oscillation can trigger the change in bubble’s volume and thus affect the translational motion. Therefore, these two types of motion for a gas bubble are not independent but coupled with each other. The movement of gas bubbles is a fundamental question in acoustic cavitation, which is widely utilized in many fields such as chemical engineering [3], hydrology [4] and biomedical ultrasound [5], [6]. A precise prediction of the bubble dynamics is the prerequisite for understanding the physical mechanisms of wave propagation in bubbly media as well as the acoustic cavitation effect.

There have been many valuable theoretical and experimental results concerning the radial oscillations for a single bubble excited by acoustic fields. The well-known Rayleigh-Plesset equation and Keller-Miksis equation provide a good description for the radial motion of a spherical bubble in incompressible and compressible fluids, respectively [7], [8]. The averaged force acting on a rigid sphere in an ideal fluid, known as the acoustic radiation force, was initially studied by King [9]. Later, Yosioka and Kawasima [10] extended the study to a compressible sphere, showing that the radiation force will be strengthened by particle’s compressibility. They also found that bubbles can exhibit more involved behaviors including erratic dancing motions and zigzag trajectories. A comprehensive study on the radiation force exerted on pulsating bubbles in a stationary field, normally called Bjerknes forces, was presented by Crum [2], who used the pressure gradient of wave fields to derive the expression of Bjerknes force [11]. It was Bleich [12] who took the lead in studying the translational motion of gas bubbles. In his work, the host fluid was assumed to be non-viscous and all the nonlinear effects of the bubble dynamics were neglected for simplicity. Watanabe and Kukita [1] first solved the equations of radial and translational motions simultaneously. The irregular translation motions for a gas bubble were predicted and a classified discussion on its dynamic behaviors depending on the bubble size was provided in their work. Later, a modified theory taking into account of the liquid compressibility was derived by Doinikov [13] using the Lagrangian formalism, which shows that any bubble can oscillate irregularly once driven by a sufficiently high pressure regardless of its initial size. The same author also studied the nonlinear coupling between volume pulsation, translational motion and shape modes of an oscillating bubble, in which all shape modes are considered without any limitations imposed on natural frequencies [14], [15]. Sadeghy and Shamekhi [16] first evaluated the effects of fluid’s elasticity on the bubble dynamics in the translational direction.

More recently, further improvement has been made for the model describing the motions of ultrasound-exerted gas bubbles both theoretically and experimentally. Cui *et al*. [17] investigated the effect of ethanol on the radial and translational motions of a levitated cavitation bubble. Melnikov [18] demonstrated that stochastic pulsations of the bubble radically change the form of its dynamic equations. Sugita *et al.* [19] experimentally extended the classical theory of Bjerknes on a single bubble to an oscillating bubble cluster in a stationary acoustic field. Ma and Chen [20] numerically studied the dynamic response of a translational bubble in a strong acoustic field as well as its influences on the cavitation effect. A trajectory observation performed by Jiao *et al.* [21] shows that the history force exhibits different behaviors at low and high pressures. Zhang *et al.* [22] derived the dynamic equation of a gas bubble in a micro-cavity. The accuracies of the time-resolved and time-averaged methods were compared by Klapcsik and Hegedus [23], who found that the former one is a preferable choice for transient waves. Wang *et al.* [24] studied the transition mechanisms of the translational motion of bubbles caused by the harmonic, subharmonic resonance and chaos.

A comprehensive review of the current references indicates that the ambient acoustic field in most studies are limited to travelling or standing plane waves, in which case only the translational motion parallel to the wave vector needs to be considered. However, the acoustic waves irradiated by transducers in practical applications are often beams with concentrated energy [25]. A gas bubble submerged in such an acoustic field can experience non-zero radiation force in both the axial and transverse directions. As a typical of the beams with a finite width, Gaussian standing waves have drawn wide attention in particle manipulation applications. In view of this, the aim of this work is directed towards the analysis of the dynamic responses of a gas bubble in a Gaussian standing wave field. A coupled system of equations of radial and translational motions are derived, followed by a numerical study on the characteristics of its dynamic behaviors. The present work can also be taken as an extension of the existing references to the non-plane wave fields.

## 2 Theoretical model

**Fig. 1:**
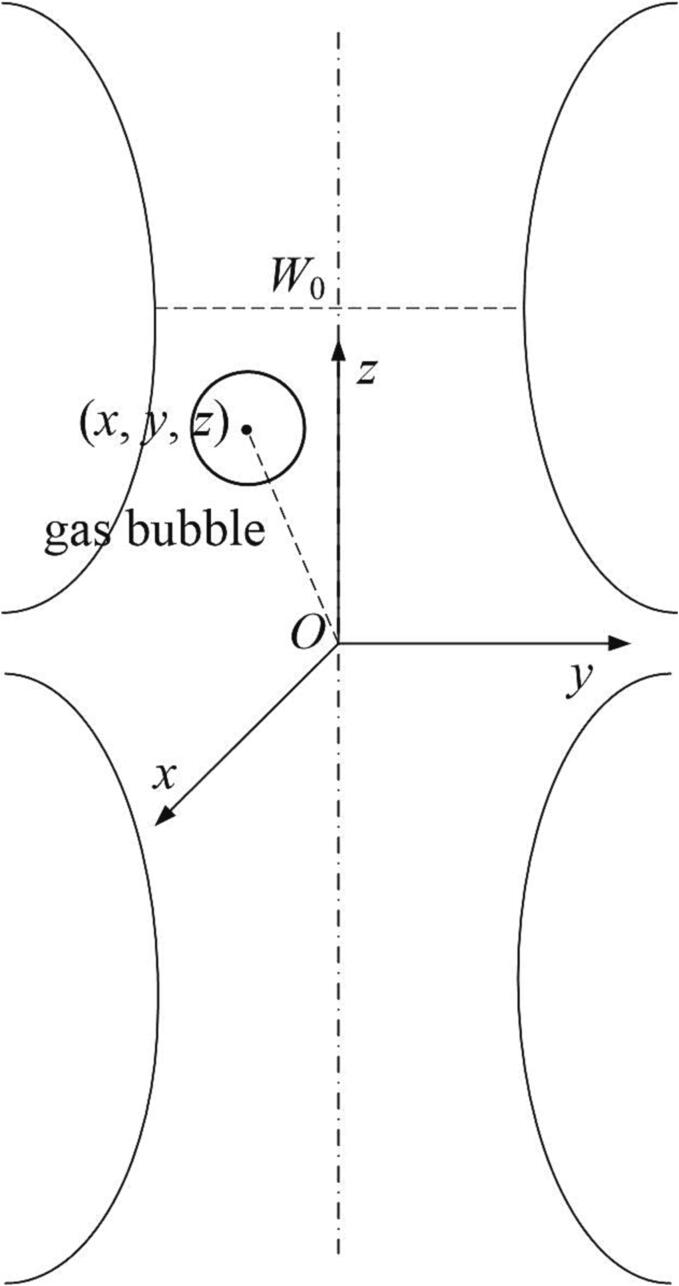
Schematic configuration of a spherical gas bubble in a Gaussian standing wave field.

Let us consider a single spherical gas bubble with the initial radius *R*_0_ surrounded by a viscous liquid medium. The mass density, the compressional wave speed, the viscosity and the surface tension coefficient of the host liquid are denoted by *ρ*, *c*, *μ* and *σ*, respectively. A monochromatic Gaussian standing wave of the angular frequency *ω* is incident upon the gas bubble, which can be regarded as the superposition of two oppositely-propagating Gaussian progressive waves. As a measurement of the focusing capability, the beam waist of the Gaussian wave is *W*_0_. The gas bubble will undergo radial oscillation and translational motion in response to the external pressure field. It is assumed that the gas bubble always maintains a spherical shape without deformation. A Cartesian coordinate system $\left(x,y,z\right)$ originates from the beam center, with the *z* axis coinciding with the beam axis. At any instant of time, the gas bubble has a radius of *R* and the center of the bubble has the coordinate of $\left(x,y,z\right)$. Fig. 1 shows the schematic diagram of the gas bubble.

For simplicity, we assume that the Gaussian standing wave satisfies the weakly focused approximation such that $kW_{0}\gg 1$. In that event, the acoustic pressure field can be simplified as [26]

(1) $$p_{\mathrm{ex}}=-A{\text{e}}^{-\frac{x^{2}+y^{2}}{W_{0}^{2}}}\sin kz\cos\omega t,$$

where *t* is time, *A* is the pressure amplitude along the beam axis, k=ω/c is the wave number in the liquid. As is shown in Eq. (1), the phase front of propagation for the Gaussian standing wave is approximately equal to that for a plane standing wave. Also, the origin coincides with the pressure node. It is presumed that the wavelength of the Gaussian standing wave is much greater than the bubble radius, so that the ambient pressure is not affected by the presence of the gas bubble. Another presumption is that the gas bubble is assumed to be located at the plane y=0 initially. Hereafter, only the translational motion in the *x* and *z* directions is required to be analyzed due to the circumferential symmetry of the acoustic field. The acoustic pressure field can be further simplified as

(2) $$p_{\mathrm{ex}}=-A{\text{e}}^{-\frac{x^{2}}{W_{0}^{2}}}\sin kz\cos\omega t.$$

### 2.1 Dynamic equations for radial oscillation

Based on the theoretical model given by Doinikov [13], the dynamic equation governing the radial oscillation for the gas bubble can be expressed as

(3) $$R\ddot{R}+\frac{3}{2}{\dot{R}}^{2}-\frac{p_{\mathrm{sc}}}{\rho }=\frac{{\dot{x}}^{2}+{\dot{z}}^{2}}{4},$$

where the overdot denotes the time derivative, the detailed expression of the pressure *p*_*sc*_ is given by

(4) $$p_{\mathrm{sc}}=\left(P_{0}+\frac{2\sigma }{R_{0}}-P_{v}\right){\left(\frac{R_{0}}{R}\right)}^{3\gamma }-\frac{2\sigma }{R}-\frac{4\mu \dot{R}}{R}+P_{v}-P_{0}-P_{\mathrm{ex}},$$

where *P*_0_ is the hydrostatic pressure, *P_v_* is the saturated vapor pressure, *γ* is the polytropic exponent of the gas.

Note that the left-hand side of Eq. (3) is simply the classical Rayleigh-Plesset equation describing the radial oscillation without considering the translational motion. The right-hand side of Eq. (3) reflects the effect of translational motion on the radial oscillation. Using the Lagrangian formalism, Doinikov [13] first proved that this feedback term is of great importance especially for high-intensity pressure fields. Since the gas bubble moves in both the axial and transverse directions, this term contains the *x* and *z* components of the translational velocity. It is also worth mentioning that the Rayleigh-Plesset equation only holds up when the velocity of radial oscillation is relatively low compared with the sound speed. For large forcing amplitudes, the left-hand side of Eq. (3) must be replaced by the Keller-Miksis equation with the right-hand side left untouched, which is expressed as [8]

(5) $$\left(1-\frac{\dot{R}}{c}\right)R\ddot{R}+\left(\frac{3}{2}-\frac{\dot{R}}{2c}\right){\dot{R}}^{2}-\frac{1}{\rho }\left(1+\frac{\dot{R}}{c}+\frac{R}{c}\frac{d}{\mathrm{dt}}\right)p_{\mathrm{sc}}=\frac{{\dot{x}}^{2}+{\dot{z}}^{2}}{4}.$$

### 2.2 Dynamic equations for translational motion

As for the translational motion of the gas bubble, force analysis is required to be performed in both the axial and transverse directions. All the forces acting on the gas bubble in the *z* direction include the gravitational force, the acoustic radiation force induced by the pressure gradient, namely the primary Bjerknes force *F_pr_*_,_*_z_*, the buoyant force *F_bu_* and the viscous drag force *F_vis_*_,_*_z_*. Besides, considering that the density of gas is much lower than that of liquid, the virtual mass effect must be taken into account, which can be equated to the virtual mass force *F_vir_*_,_*_z_*. A detailed analysis of each force is given as follows.

The gravitational force and the buoyant force of the gas bubble are expressed, respectively as

(6) $$G=-m_{b}g,$$

(7) $$F_{\mathrm{bu}}=\rho Vg,$$

where *m_b_* is the mass of the gas inside the bubble, *g* is the local gravitational acceleration, $V=4\pi R^{3}/3$ is the volume of the gas bubble. The primary Bjerknes force is given by

(8) $$F_{\mathrm{pB},z}=-V\frac{dp_{\mathrm{ex}}}{\mathrm{dz}}.$$

The viscous drag force is defined as [27]

(9) $$F_{\mathrm{vis},z}=-12\pi \mu R\left(\dot{z}-v_{\mathrm{ex},z}\right),$$

where *v_ex_*_,_*_z_* is the *z* component of the particle velocity generated by the acoustic pressure field, which is calculated from Eq. (2) to be

(10) $$v_{\mathrm{ex},z}=\frac{A}{\rho c}{\text{e}}^{-\frac{x^{2}}{W_{0}^{2}}}\cos kz\sin\omega t.$$

The virtual mass force is described as

(11) $$F_{\mathrm{vir},z}=-\frac{1}{2}\rho \frac{d}{\mathrm{dt}}\left[V\left(\dot{z}-v_{\mathrm{ex},z}\right)\right],$$

Based on the Newton’s second law, the equation of motion in the *z* direction can be written as

(12) $$m_{b}\ddot{z}=-m_{b}g-V\frac{dp_{\mathrm{ex}}}{\mathrm{dz}}-\frac{1}{2}\rho \frac{d}{\mathrm{dt}}\left[V\left(\dot{z}-v_{\mathrm{ex},z}\right)\right]+\rho Vg-12\pi \mu R\left(\dot{z}-v_{\mathrm{ex},z}\right),$$

Considering that the mass of the bubble is negligible compared with the virtual mass, the inertia and gravity terms can be omitted, further simplifying Eq. (12) as

(13) $$-V\frac{dp_{\mathrm{ex}}}{\mathrm{dz}}-\frac{1}{2}\rho \frac{d}{\mathrm{dt}}\left[V\left(\dot{z}-v_{\mathrm{ex},z}\right)\right]+\rho Vg-12\pi \mu R\left(\dot{z}-v_{\mathrm{ex},z}\right)=0.$$

The translational motion in the *x* direction is driven by the primary Bjerknes force *F_pr_*_,_*_z_*, the viscous drag force *F_vis_*_,_*_z_* and the virtual mass force *F_vir_*_,_*_z_*. In analogy to the force analysis above, it is easy to obtain their expressions as

(14) $$F_{\mathrm{pB},x}=-V\frac{dp_{\mathrm{ex}}}{\mathrm{dx}},$$

(15) $$F_{\mathrm{vis},x}=-12\pi \mu R\left(\dot{x}-v_{\mathrm{ex},x}\right),$$

(16) $$F_{\mathrm{vir},x}=-\frac{1}{2}\rho \frac{d}{\mathrm{dt}}\left[V\left(\dot{x}-v_{\mathrm{ex},x}\right)\right].$$

where *v_ex_*_,_*_x_* is the *x* component of the particle velocity generated by the acoustic pressure field, which can also be calculated from Eq. (2) as

(17) $$v_{\mathrm{ex},x}=-\frac{A}{\rho \omega }{\text{e}}^{-\frac{x^{2}}{W_{0}^{2}}}\frac{2x}{W_{0}^{2}}\sin kz\sin\omega t.$$

Hence, the equation of motion in the *x* direction can be expressed based on the Newton’s second law as

(18) $$m_{b}\ddot{x}=-V\frac{dp_{\mathrm{ex}}}{\mathrm{dx}}-\frac{1}{2}\rho \frac{d}{\mathrm{dt}}\left[V\left(\dot{x}-v_{\mathrm{ex},x}\right)\right]-12\pi \mu R\left(\dot{x}-v_{\mathrm{ex},x}\right).$$

Likewise, omission of the inertia and gravity terms yields a simplified form of Eq. (18) as

(19) $$-V\frac{dp_{\mathrm{ex}}}{\mathrm{dx}}-\frac{1}{2}\rho \frac{d}{\mathrm{dt}}\left[V\left(\dot{x}-v_{\mathrm{ex},x}\right)\right]-12\pi \mu R\left(\dot{x}-v_{\mathrm{ex},x}\right)=0.$$

Eq. (13), (19) govern the translational motion in the *z* and *x* directions for the gas bubble, respectively. Inspection of Eq. (4), (13), (19) or Eq. (5), (13), (19) indicates that the radial and translational motions are coupled through the volume of the gas bubble.

## 3 Results and discussion

Numerical computations are performed based on the theoretical analysis above to investigate the dynamic behaviors for a single gas bubble in a Gaussian standing wave field. The ordinary differential equations given by Eq. (4), (13), (19) or Eq. (5), (13), (19) are solved through the fourth-order Runge-Kutta method. For the radial oscillation of the gas bubble, the time step of the numerical solution is set to 1/1000 of the acoustic period to avoid missing details of the radius variation. When studying the translational motion of the gas bubble, however, we increase the time step to the acoustic period as thousands of acoustic cycles are going to be investigated. The values for liquid density, wave speed, surface tension coefficient, hydrostatic pressure, liquid viscosity and specific heat ratio are set to $\rho =1000\;\text{kg}/{\text{m}}^{3}$, c=1500m/s, σ=0.072N/m, $P_{0}=1.013\times 10^{5}\;\text{Pa}$, $\mu =1.0\times 10^{-3}\;\text{Pa}\cdot \text{s}$, γ=1.4, respectively so as to simulate an air bubble immersed in water at atmospheric conditions.

**Fig. 2:**
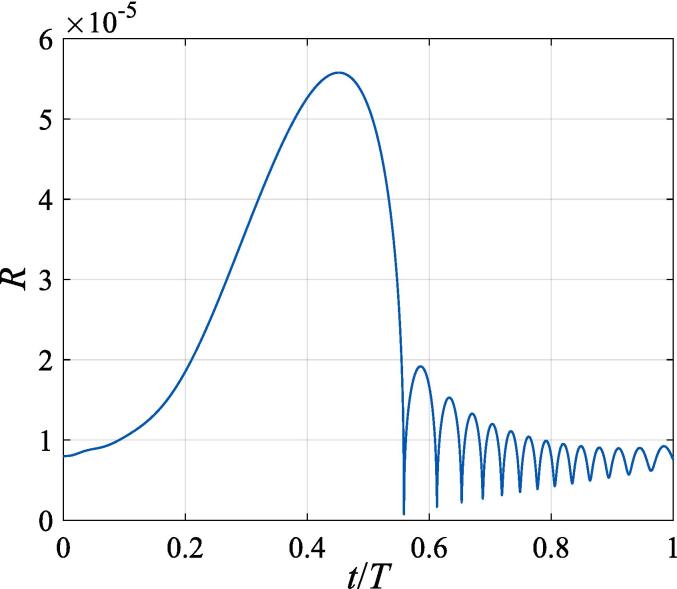
Radial oscillation of a gas bubble in a plane standing wave field with $A=1.32\times 10^{5}Pa,f=20kHz,R_{0}=8\mathrm{\mu m}$.

To verify the validity of the present theorem, the radial response of a gas bubble is first studied in a plane standing wave field by setting the beam waist to infinity, with the driving frequency f=20kHz, the pressure amplitude $A=1.32\times 10^{5}\;\text{Pa}$ and the initial radius of the bubble $R_{0}=8\mathrm{\mu m}$. At t=0, the bubble is located at a distance of 1/50λ from the pressure antinode without any initial velocity. Fig. 2 displays the instant bubble radius versus time within the first acoustic cycle. As is shown in the figure, the variation of bubble radius can be generally divided into three processes including expansion, contraction and oscillation. The simulated plot consists well with the result given in Ref. [20] except for some minor differences due to negligence of the saturated vapor pressure in that work.

In all of the following computations, the gas bubble has an initial radius of $R_{0}=10\mathrm{\mu m}$ and no initial velocity in both the radial and translational directions. The resonance frequency of the gas bubble can be obtained from the linear oscillation theory, which is given by [28]

(20) $$f_{\mathrm{res}}=\frac{1}{2\pi R_{0}}\sqrt{\frac{3\gamma P_{0}}{\rho }+\frac{2\left(3\gamma -1\right)\sigma }{\rho R_{0}}}=3.42\times 10^{5}\;\text{Hz}\text{.}$$

### 3.1 Computational results of radial oscillation

**Fig. 3:**
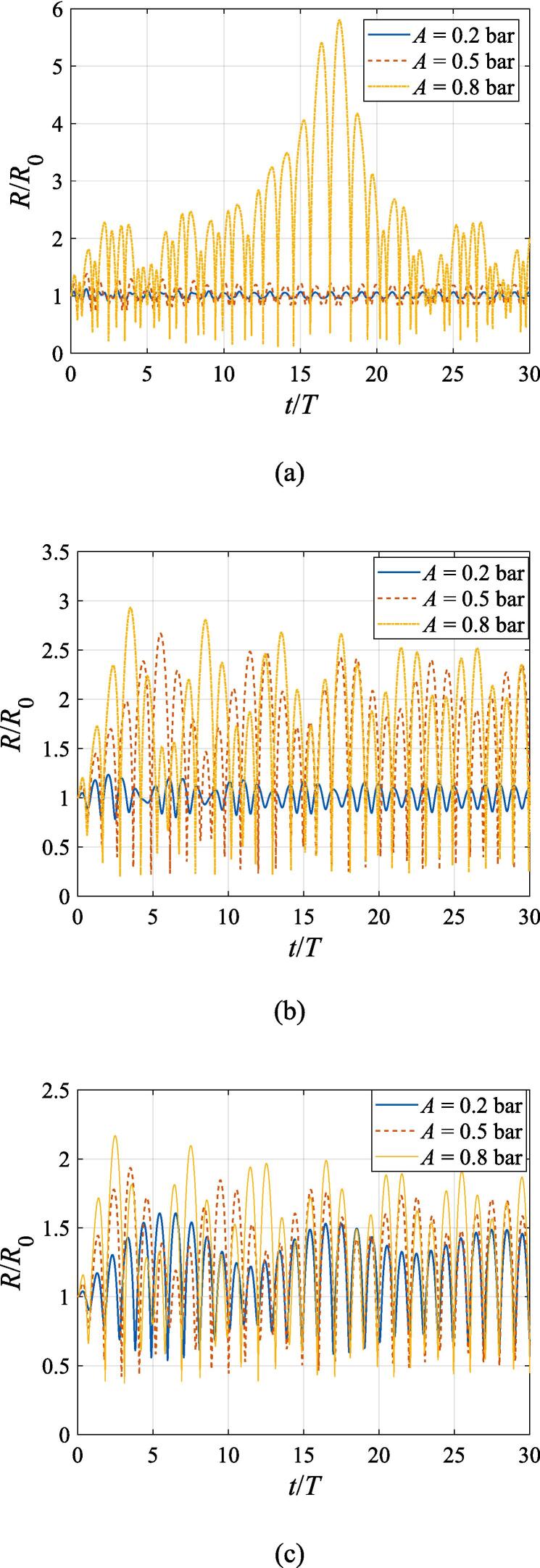

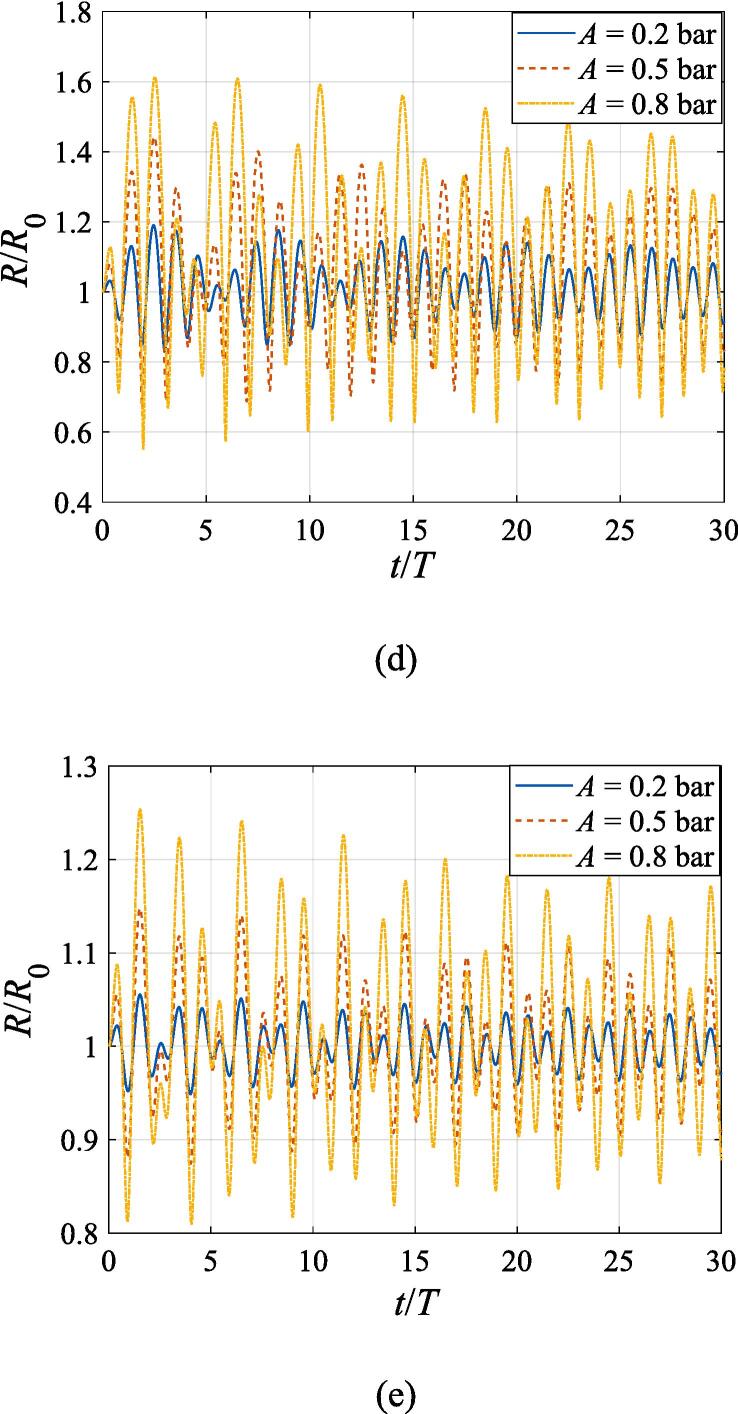
Radial oscillation of a gas bubble in a Gaussian standing wave field for different pressure amplitudes with $z_{0}=\lambda /4,x_{0}=0,W_{0}=\text{3}\lambda$. The driving frequencies in panel (a), (b), (c), (d) and (e) are 137 kHz, 274 kHz, 342 kHz, 411 kHz and 547 kHz, respectively.

**Fig. 4:**
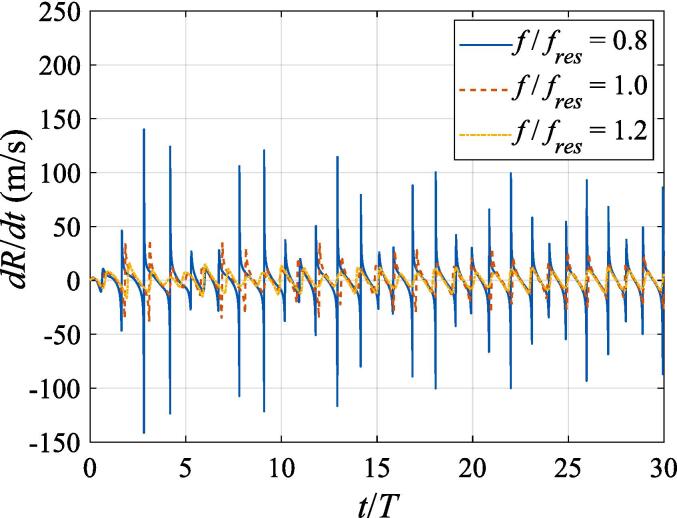
Radial velocity of a gas bubble in a Gaussian standing wave field for different driving frequencies with $z_{0}=\lambda /4,x_{0}=0,A=0.8\;\text{bar,}\;W=\text{3}\lambda$.

The radial oscillation for the gas bubble is first examined in this section. Fig. 3 shows the instant bubble radius versus time during the first thirty acoustic cycles. The bubble radius *R* denoted by the vertical axis is normalized by the initial radius *R*_0_, and the time *t* denoted by the horizontal axis is normalized by the acoustic period *T*. Initially, the gas bubble is at $z_{0}=\lambda /4$ and $x_{0}=0$. This setting of parameters corresponds to the first pressure antinode along the beam axis, where the acoustic pressure reaches its highest value and the axial and transverse radiation forces both vanish due to symmetry. While the buoyant force is nonzero in the *z* direction, the calculating period is so short that the translational displacement can be neglected for the bubble. The beam waist of the Gaussian standing wave is set to $W_{0}=3\lambda$ and the driving frequencies in panel (a), (b), (c), (d) and (e) are f=137kHz, 274 kHz, 342 kHz, 411 kHz, 547 kHz, equal to $f=0.4f_{\mathrm{res}},0.8f_{\mathrm{res}},f_{\mathrm{res}},1.2f_{\mathrm{res}},1.6f_{\mathrm{res}}$, respectively. For each driving frequency, the numerical computations are performed at A=0.2bar, 0.5 bar, 0.8 bar, respectively. After a few periods of transient process, the gas bubble oscillates around its equilibrium radius with a decaying amplitude due to viscosity. However, since the expansion and contraction processes are not exactly symmetrical with respect to its equilibrium radius, nonlinearity of the bubble oscillation is also clearly exhibited in the simulated results. As the pressure amplitude rises, the amplitude of the radial oscillation also increases significantly amid the steady-state response. Generally, the oscillation amplitude grows as the driving frequency approaches the resonance frequency. However, the maximum amplitude occurs at $f=0.8f_{\mathrm{res}}$ (Fig. 3(b)) rather than $f=f_{\mathrm{res}}$ (Fig. 3(c)). This phenomenon can also be attributed to nonlinearity of the radial dynamic equations. Since the gas bubble is assumed to be located at the beam axis without initial velocity, we can safely arrive at the conclusion that the radial oscillation follows the same rules as that in a plane standing wave field. As is shown in Fig. 4, the radial velocity of the gas bubble at A=0.8bar around the resonance frequency is no more than 150m/s, which is much smaller than the sound speed of the surrounding liquid. Hence, it is reasonable for us to utilize Eq. (3) to describe the radial oscillation of the gas bubble.

**Fig. 5:**
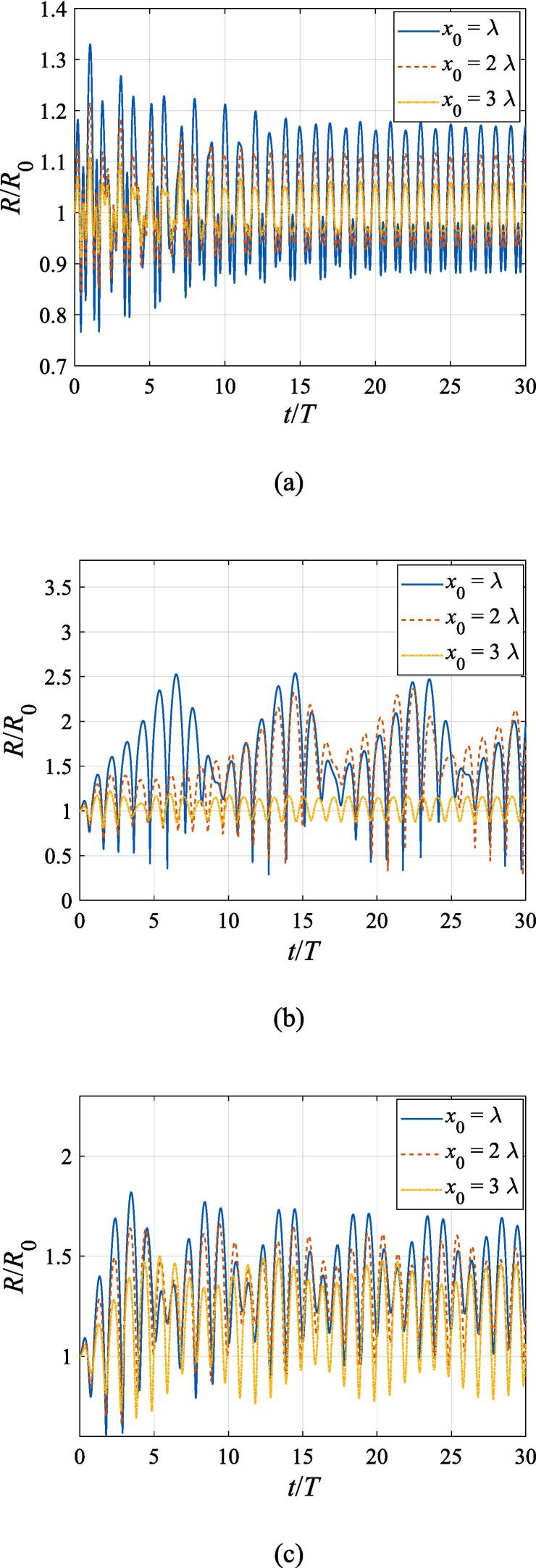

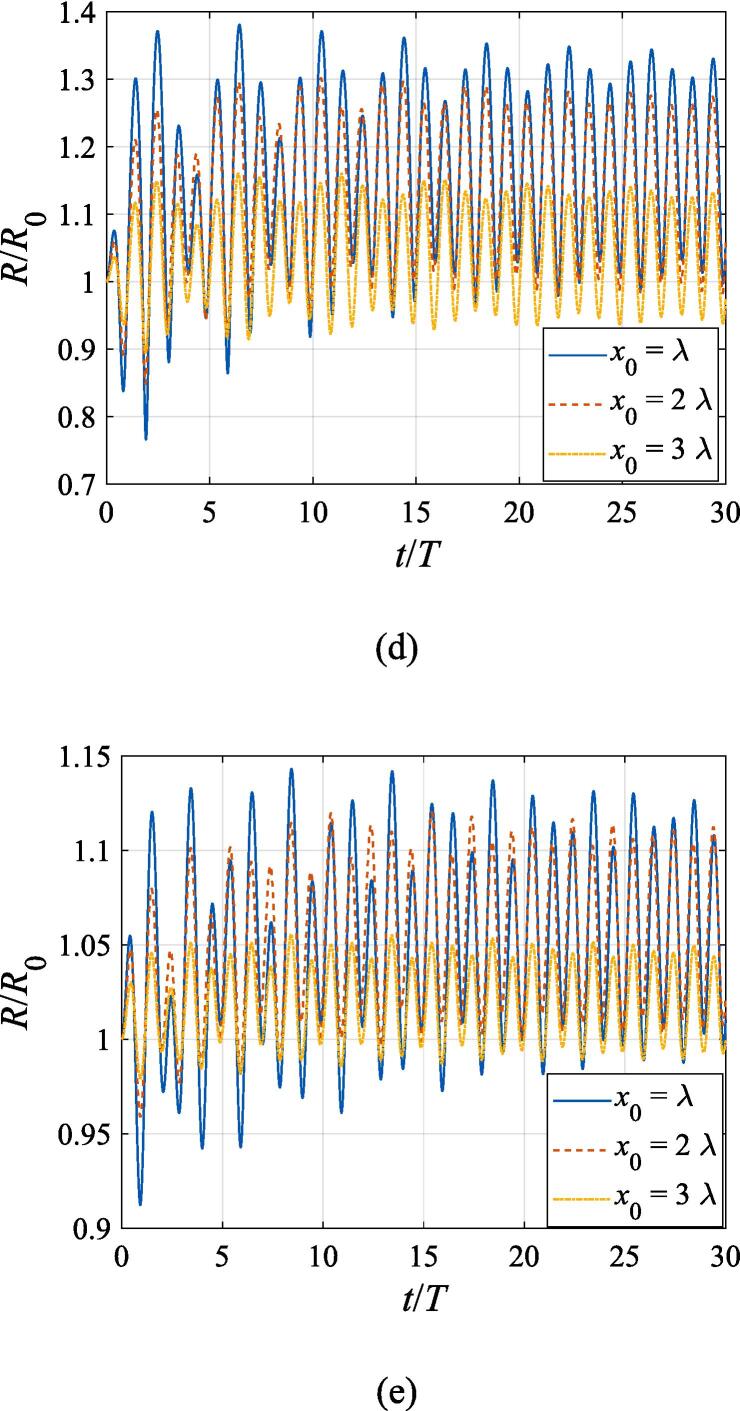
Radial oscillation of a gas bubble in a Gaussian standing wave field for different off-axial distances with $z_{0}=\lambda /4,W_{0}=3\lambda ,A=\text{0.5 bar}$. The driving frequencies in panel (a), (b), (c), (d) and (e) are 137 kHz, 274 kHz, 342 kHz, 411 kHz and 547 kHz, respectively.

Unlike the case of plane wave incidence, the dynamic behaviors for a gas bubble vary at different transverse positions. The normalized bubble radius versus the normalized time plots for the gas bubble are displayed in Fig. 5 within the first five acoustic cycles. The beam waist and the driving frequencies of the Gaussian standing wave in panel (a), (b), (c), (d) and (e) remain unchanged, while the pressure amplitude is fixed to A=0.5bar amid the numerical computation. With the initial *z* coordinate still satisfying $z_{0}=\lambda /4$, the initial *x* coordinate is set to $x_{0}=\lambda ,\;\text{2}\lambda ,\;\text{3}\lambda$, respectively for each panel. It is shown that the radial oscillation generally possesses the same growing trend as those in Fig. 3. The oscillation amplitude, however, is lowered due to the damping effect of acoustic power in the off-axial configuration. A brief quantitative analysis can also be given on the attenuation of the oscillation amplitude. For instance, the pressure amplitude at $x_{0}=3\lambda$ is equal to e^-1^ of its maximum value along the beam axis based on Eq. (2). Therefore, the amplitudes of the forced radial vibration also approximately decrease to e^-1^ of the counterparts in Fig. 3.

### 3.2 Computational results of translational motion

**Fig. 6:**
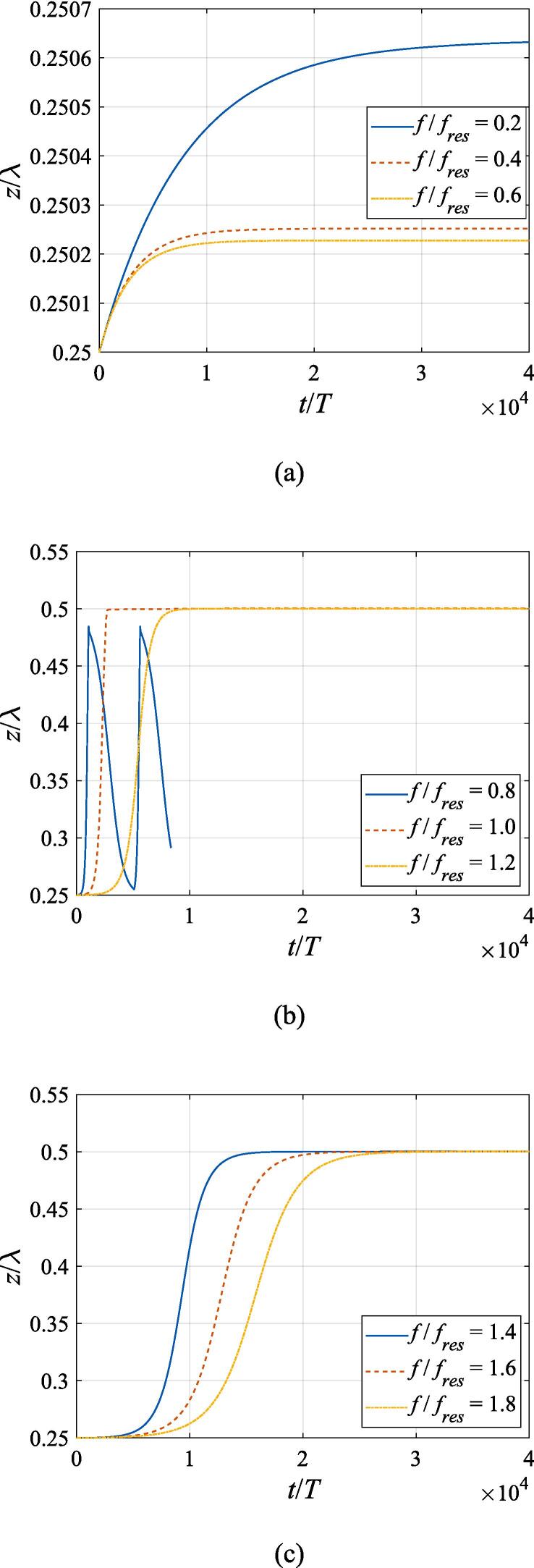
Translational motion of a gas bubble in a Gaussian standing wave field for different driving frequencies with $z_{0}=\lambda /4,x_{0}=0,W_{0}=3\lambda ,A=0.5\;\text{bar}$. The driving frequencies are 68.4 kHz, 137 kHz, 205 kHz in panel (a), 274 kHz, 342 kHz, 411 kHz in panel (b) and 479 kHz, 547 kHz, 616 kHz in panel (c), respectively.

In this section, the translational motion for the gas bubble is going to be investigated based on the dynamic equations above. Considering that the translational velocity is much lower than the radial one, the system of equations is required to be solved in a much longer period so as to give a more comprehensive display of the bubble dynamics. Fig. 6 presents the translational motion of the gas bubble subject to a Gaussian standing wave field with $W_{0}=3\lambda$. The bubble is positioned at $z_{0}=\lambda /4$ and $x_{0}=0$ without any initial velocity at t=0. Therefore, its trajectory is confined to the *z* axis due to symmetry. The pressure amplitude is fixed to A=0.5bar. The driving frequency in panel (a) is set to f=68.4kHz,137kHz, 205 kHz, corresponding to $f=0.2f_{\mathrm{res}},0.4f_{\mathrm{res}},\;\text{0.6}f_{\mathrm{res}}$, respectively, which are much lower than the resonance frequency. The driving frequency in panel (b) is set to f=274kHz, 342 kHz, 411 kHz, corresponding to $f=0.8f_{\mathrm{res}},f_{\mathrm{res}},\;\text{1.2}f_{\mathrm{res}}$, respectively, which are near the resonance frequency. The driving frequency in panel (c) is set to f=479kHz, 547 kHz, 616 kHz, corresponding to $f=1.4f_{\mathrm{res}},\;\text{1.6}f_{\mathrm{res}},\;\text{1.8}f_{\mathrm{res}}$, respectively, which are much higher than the resonance frequency. According to the theory in former references [1], [13], when the driving frequency is lower than the resonance frequency, bubbles will move to pressure antinodes and gather there. However, the buoyant force is included in our discussion and will drive the gas bubble to ascend in the + *z* direction as is shown in Fig. 6(a). Once leaving the pressure antinode, the bubble will be slowed down by the primary Bjerknes force and viscous drag force. Finally, it reaches a new equilibrium position above the pressure antinode. A Gaussian standing wave with a higher driving frequency induces a stronger primary Bjerknes force and thus the translational motion will be stopped much earlier. It is also noted that the displacement of the gas bubble is no more than 1/1000 of the wavelength, which is completely negligible for a naked-eye observation. As the driving frequency grows to 0.8 *f_res_* in Fig. 6(b), however, the gas bubble will not be trapped close to the pressure antinode by the primary Bjerknes force. Instead, it moves towards the next pressure node at z=0.5λ and then executes dramatic oscillation between z=0.25λ and z=0.5λ with a period of around 5000 *T*. It has been investigated in former studies that this erratic translational motion can be ascribed to the reversal of the primary Bjerknes force due to the change of phase shift between the gas bubble and the incident pressure [13], [29]. Unfortunately, problems are encountered during the numerical calculation which only allow the bubble path to be accurately followed until about 8000 acoustic cycles. Therefore, our calculation is terminated much earlier at $f=0.8f_{\mathrm{res}}$ to avoid divergence. For $f=f_{\mathrm{res}}$ or $f=1.2f_{\mathrm{res}}$ in Fig. 6(b), the gas bubble is directed towards the pressure node and then settled there without any oscillation, which is in good agreement with Ref. [13]. Compared to the case of $f=f_{\mathrm{res}}$, a decrease of the primary Bjerknes force will delay the arrival time in the case of $f=1.2f_{\mathrm{res}}$. With the driving frequency further increasing to $f=1.4f_{\mathrm{res}}\text{,}\;1.6f_{\mathrm{res}},1.8f_{\mathrm{res}}$ in Fig. 6(c), the pressure node at z=0.5λ remains the stable equilibrium position for the gas bubble and the arrival time still has a positive correlation with the driving frequency.

**Fig. 7:**
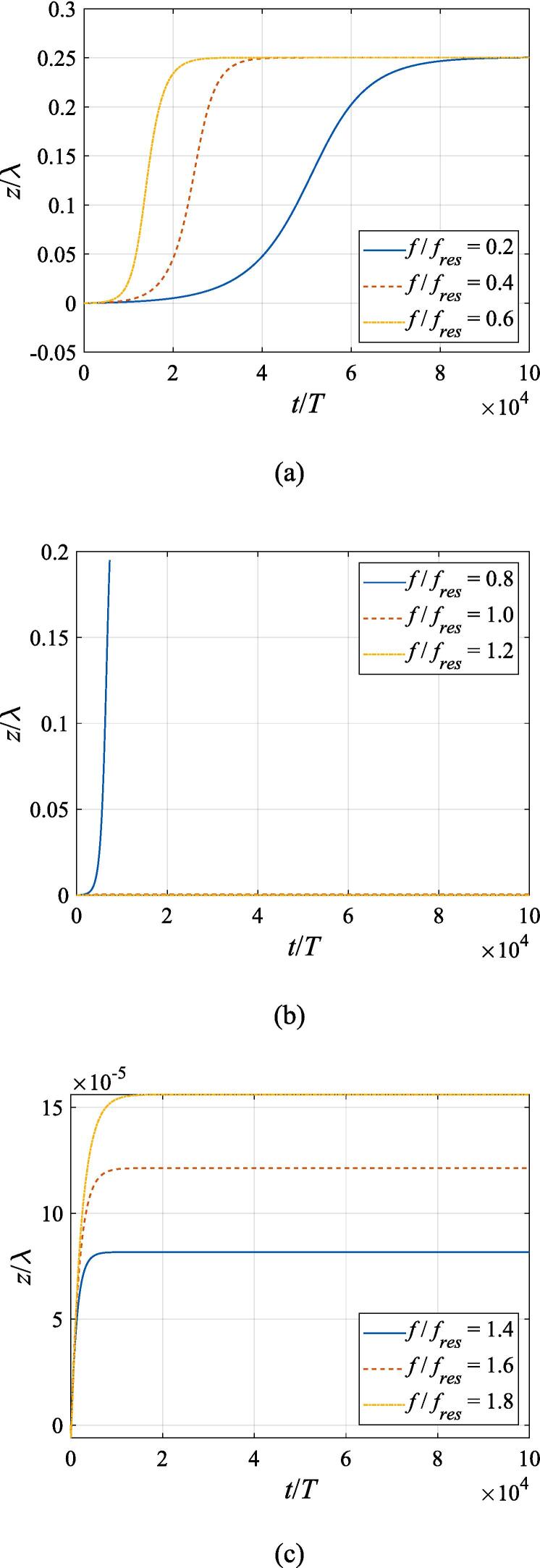
Translational motion of a gas bubble in a Gaussian standing wave field for different driving frequencies with $z_{0}=0,x_{0}=0,W_{0}=3\lambda ,A=0.5\;\text{bar}$. The driving frequencies are 68.4 kHz, 137 kHz, 205 kHz in panel (a), 274 kHz, 342 kHz, 411 kHz in panel (b) and 479 kHz, 547 kHz, 616 kHz in panel (c), respectively.

With the beam waist, pressure amplitude and driving frequencies remaining unchanged, Fig. 7 shows the translational motion for the gas bubble initially at $z_{0}=0$ and $x_{0}=0$, corresponding to the first pressure node of the Gaussian standing wave. As can be expected from the analysis above, the gas bubble in Fig. 7(a) is directed towards the pressure antinode and a pressure field with a higher driving frequency generates a larger translational velocity and an earlier arrival time. Exerted by the buoyant force, the gas bubble driven above the resonance frequency in Fig. 7(b) and (c) will move in the + *z* direction and finally reaches equilibrium very close to the pressure node, similar to the simulated plots in Fig. 6(a). When the driving frequency amounts to 0.8 *f_res_* in Fig. 7(b), the gas bubble should have ascended to the pressure antinode. Nevertheless, the numerical calculation is terminated much earlier in this case to avoid divergence.

**Fig. 8:**
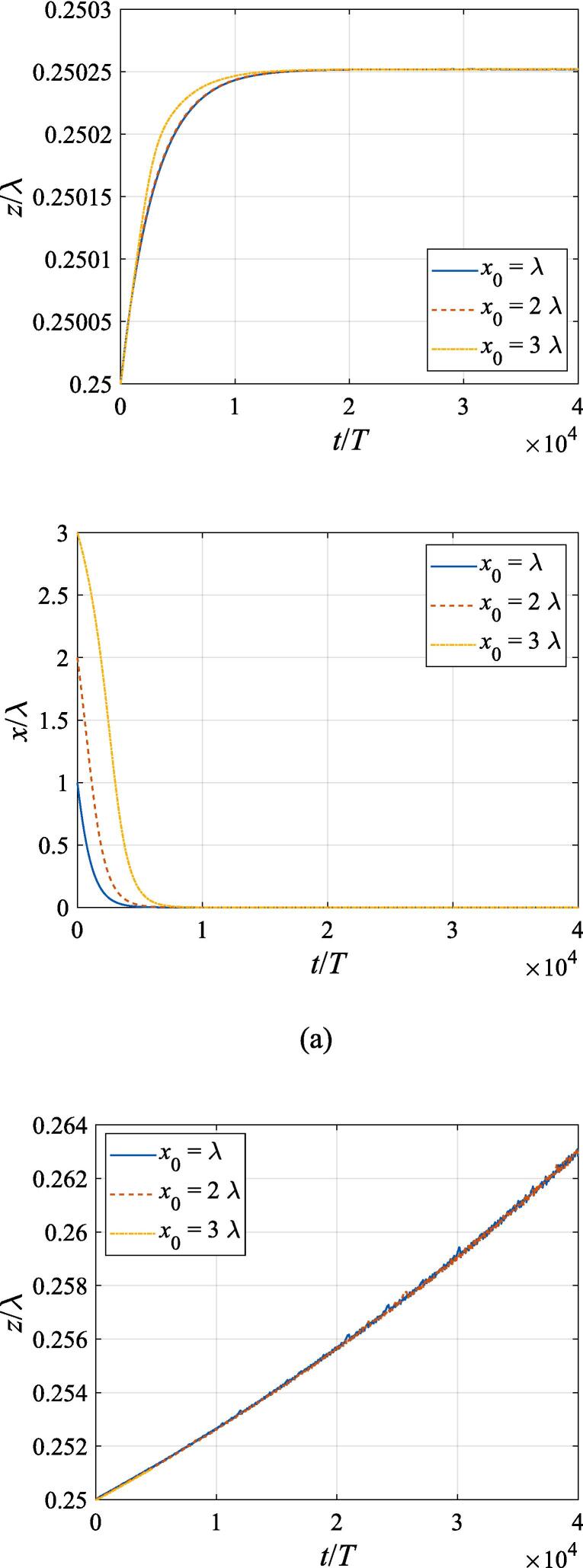

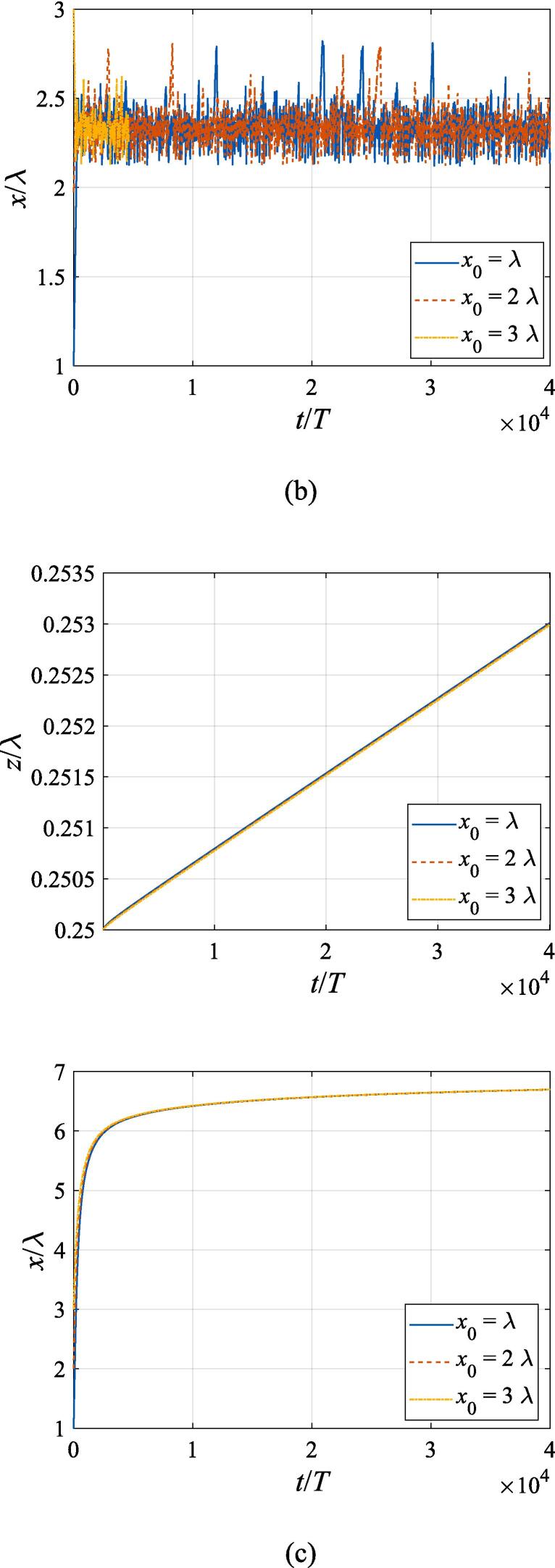

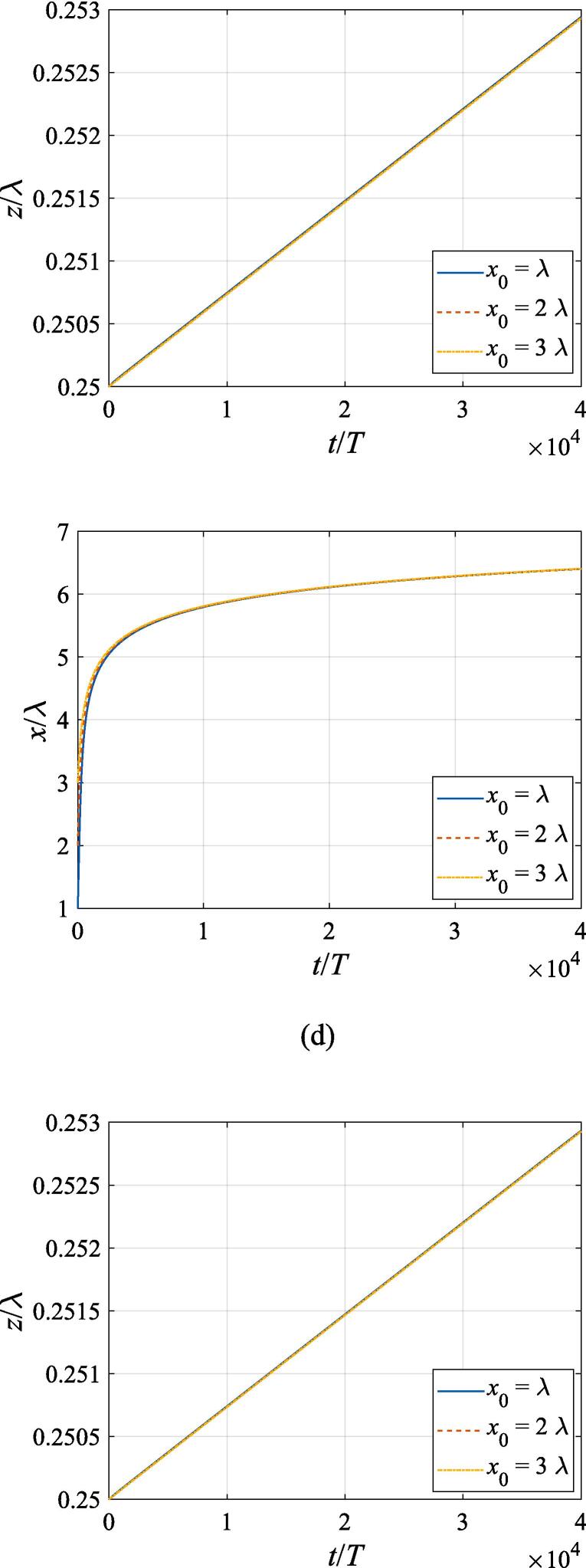

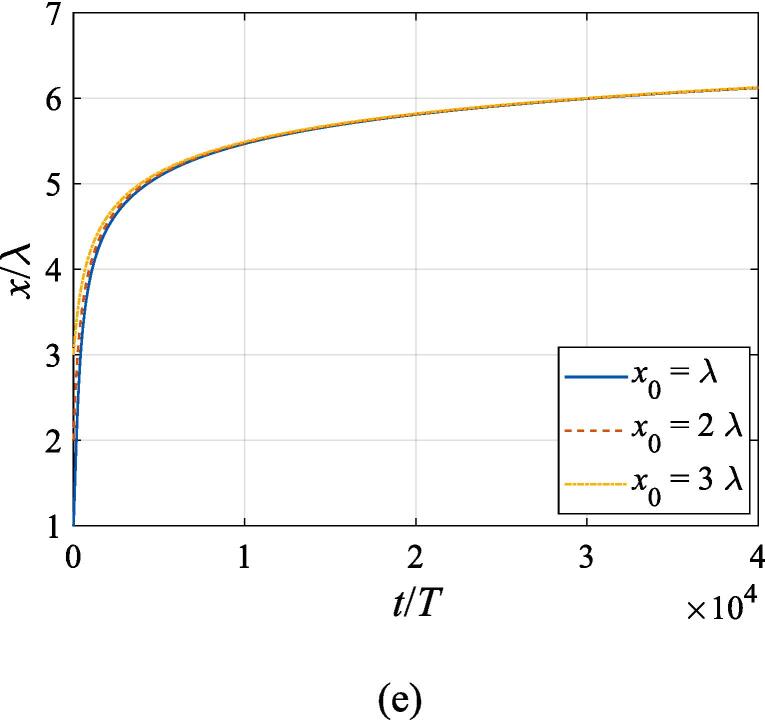
Translational motion of a gas bubble in a Gaussian standing wave field for different off-axial distances with $z_{0}=\lambda /4,W_{0}=3\lambda ,A=0.5\;\text{bar}$. The driving frequencies in panel (a), (b), (c), (d) and (e) are 137 kHz, 274 kHz, 342 kHz, 411 kHz and 547 kHz, respectively.

Fig. 8 extends the discussion above to the off-axial configuration, where the translational motion in both the *x* and *z* directions is required to be taken into account. The initial *z* and *x* coordinates are set to $z_{0}=\lambda /4$ and $x_{0}=\lambda ,2\lambda ,3\lambda$, respectively. With other physical parameters remaining the same as in Fig. 7, the cases of f=137kHz,274kHz, 342 kHz, 411 kHz, 547 kHz are investigated in panel (a), (b), (c), (d) and (e), corresponding to $f=0.4f_{\mathrm{res}},0.8f_{\mathrm{res}},f_{\mathrm{res}},1.2f_{\mathrm{res}},1.6f_{\mathrm{res}}$, respectively. At $f=0.4f_{\mathrm{res}}$ (Fig. 8(a)), the gas bubble is pulled towards the beam axis by the transverse primary Bjerknes force within 10^4^ acoustic cycles. Meanwhile, a tiny displacement in the axial direction is also observed for the bubble, which finally reaches equilibrium at the same position irrespective of its initial off-axial distance. In Fig. 8(c), (d) and (e) when the driving frequency is not less than the resonance frequency, the transverse primary Bjerknes force reverses it sign, pointing outwards from the beam axis. A combination of the radiation force and viscous drag force results in a decelerating motion in the + *x* direction. As for the *z* component, the bubble nearly makes slow uniform linear motion, indicating that equilibrium is reached in the axial direction. It is also interesting to find that, for bubbles driven above the resonance frequency, the translational motion is not significantly affected by the initial off-axial distance. Actually, in this case, the buoyant force and viscous drag force dominate the bubble dynamics. When the driving frequency is equal to 0.8 *f_res_* (Fig. 8(b)), the problem of divergence occurs only at $x_{0}=3\lambda$, forcing us to terminate the calculation ahead of time. Whatever the initial off-axial distance is, the gas bubble will quickly move to $x_{0}=2.25\lambda$ and exhibit sophisticated oscillation about it resulting from nonlinearity of the dynamics equations.

**Fig. 9:**
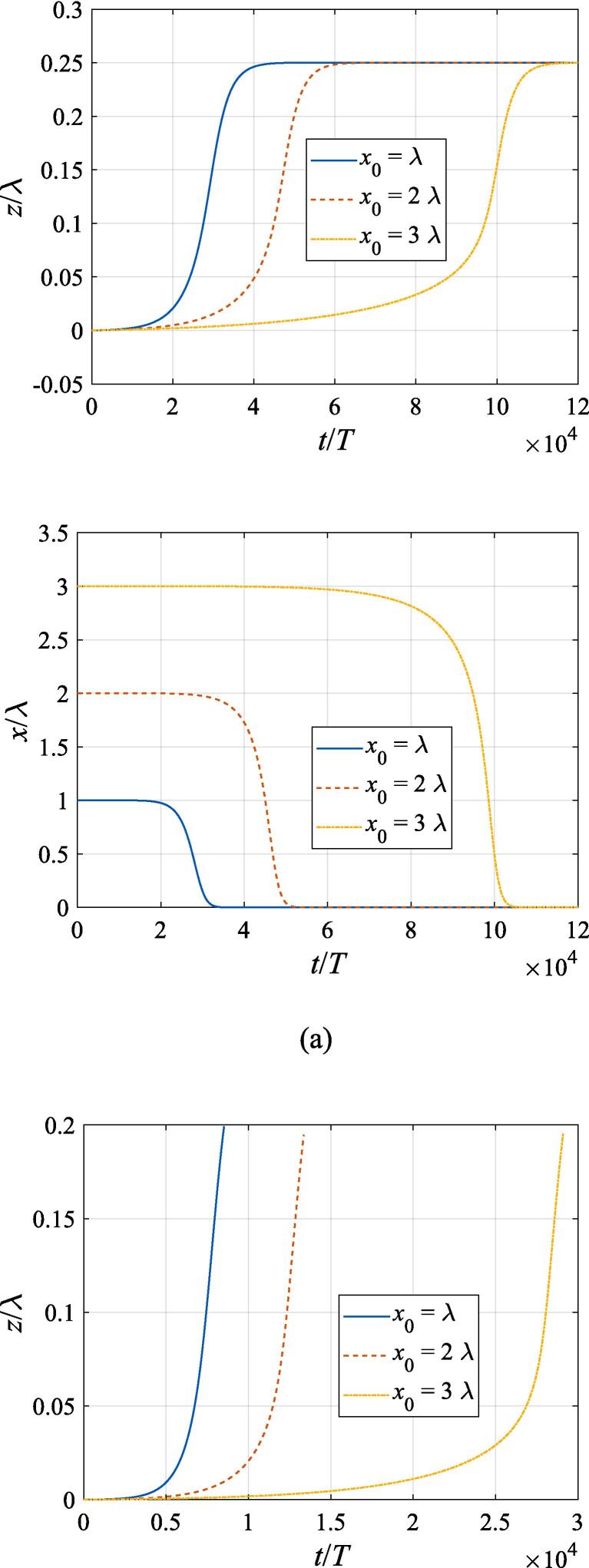

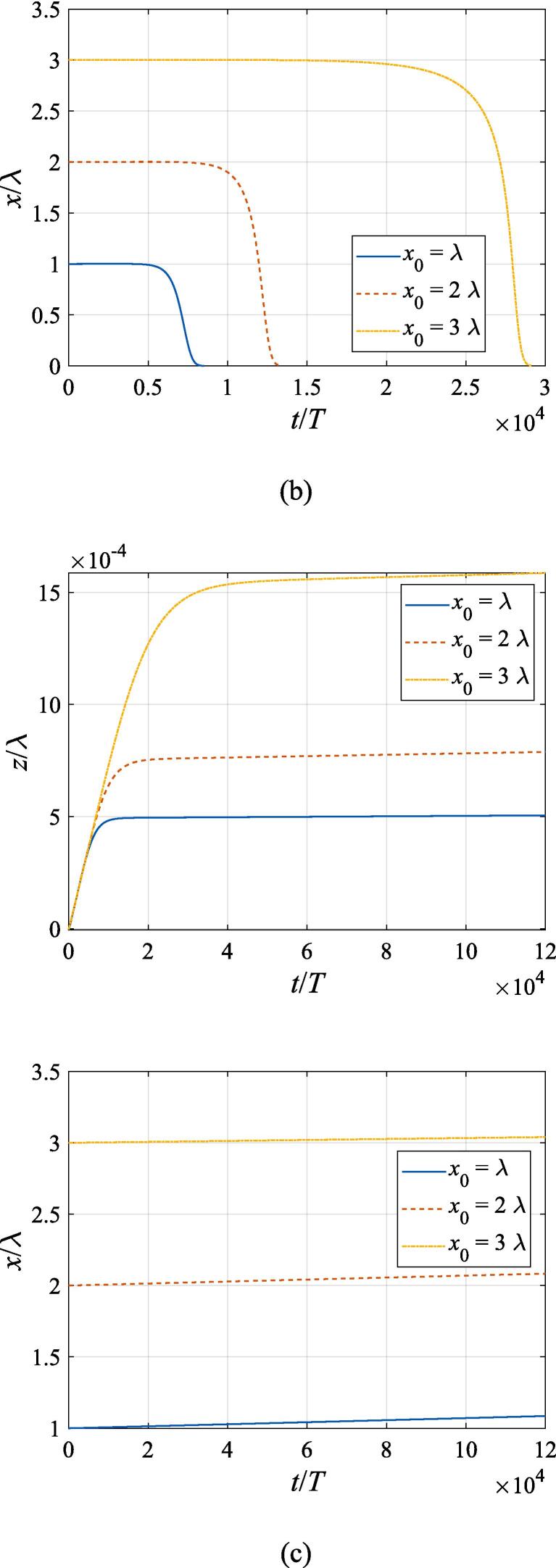

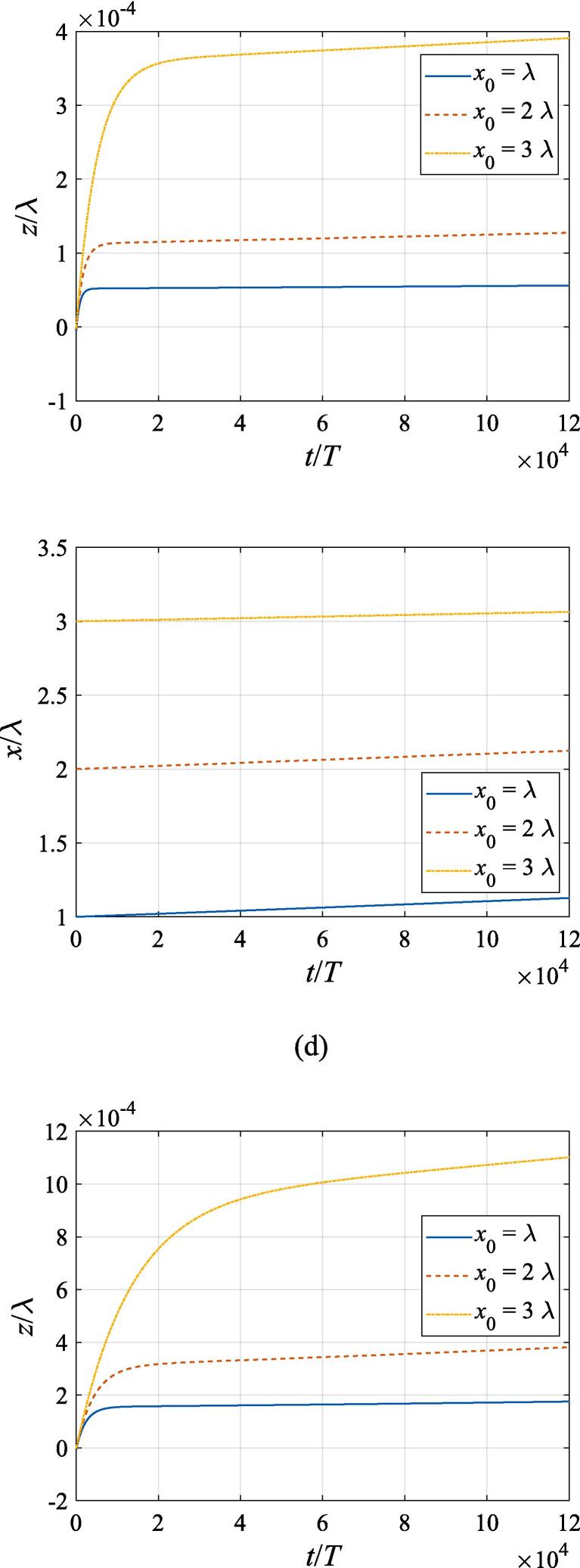

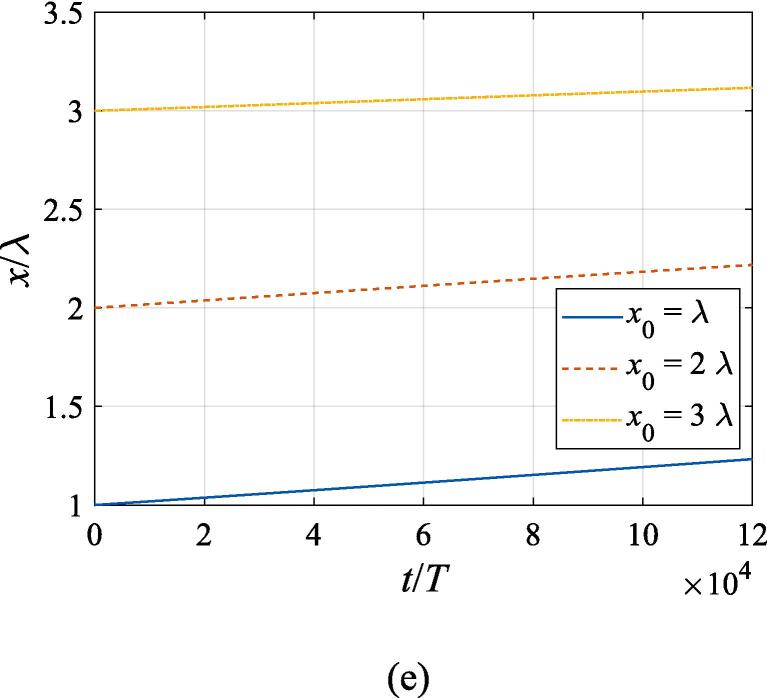
Translational motion of a gas bubble in a Gaussian standing wave field for different off-axial distances with $z_{0}=0,W_{0}=3\lambda ,A=0.5\;\text{bar}$. The driving frequencies in panel (a), (b), (c), (d) and (e) are 137 kHz, 274 kHz, 342 kHz, 411 kHz and 547 kHz, respectively.

Similarly, we switch to the study of the bubble dynamics when it is located at $z_{0}=0$ initially corresponding to the pressure node. As is shown in Fig. 9(a), the gas bubble driven much below the resonance frequency makes for the first pressure antinode under the primary Bjerknes force. It takes the bubble a longer time to reach equilibrium with increasing *x*_0_ values due to attenuation of the acoustic power. As the bubble approaches z=0.25λ, it also moves towards the beam axis driven by the transverse primary Bjerknes force, with the transverse velocity synchronously changing with the axial one. In Fig. 9(c), (d) and (e) when the bubble is driven above the resonance frequency, since the axial primary Bjerknes force almost vanishes, only a small perturbation of its *z* coordinate occurs due to presence of the buoyant force and viscous drag force. Note that the transverse radiation force reverses its direction again, driving the bubble outwards from the beam axis at a low speed. The time scale at $f=0.8f_{\mathrm{res}}$ in Fig. 9(b), though much shortened due to divergence of calculation, is enough for us to observe the entire process of the bubble returning the beam axis.

### 3.3 Effects of the beam waist on radial and translational motions

**Fig. 10:**
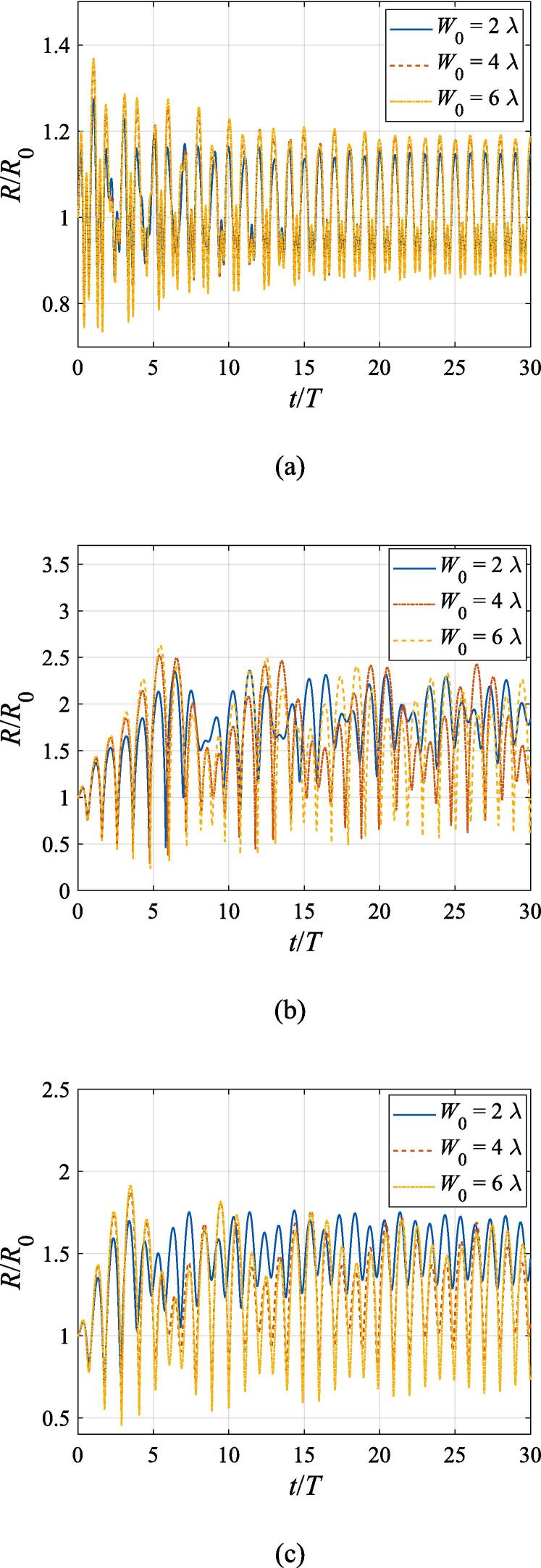

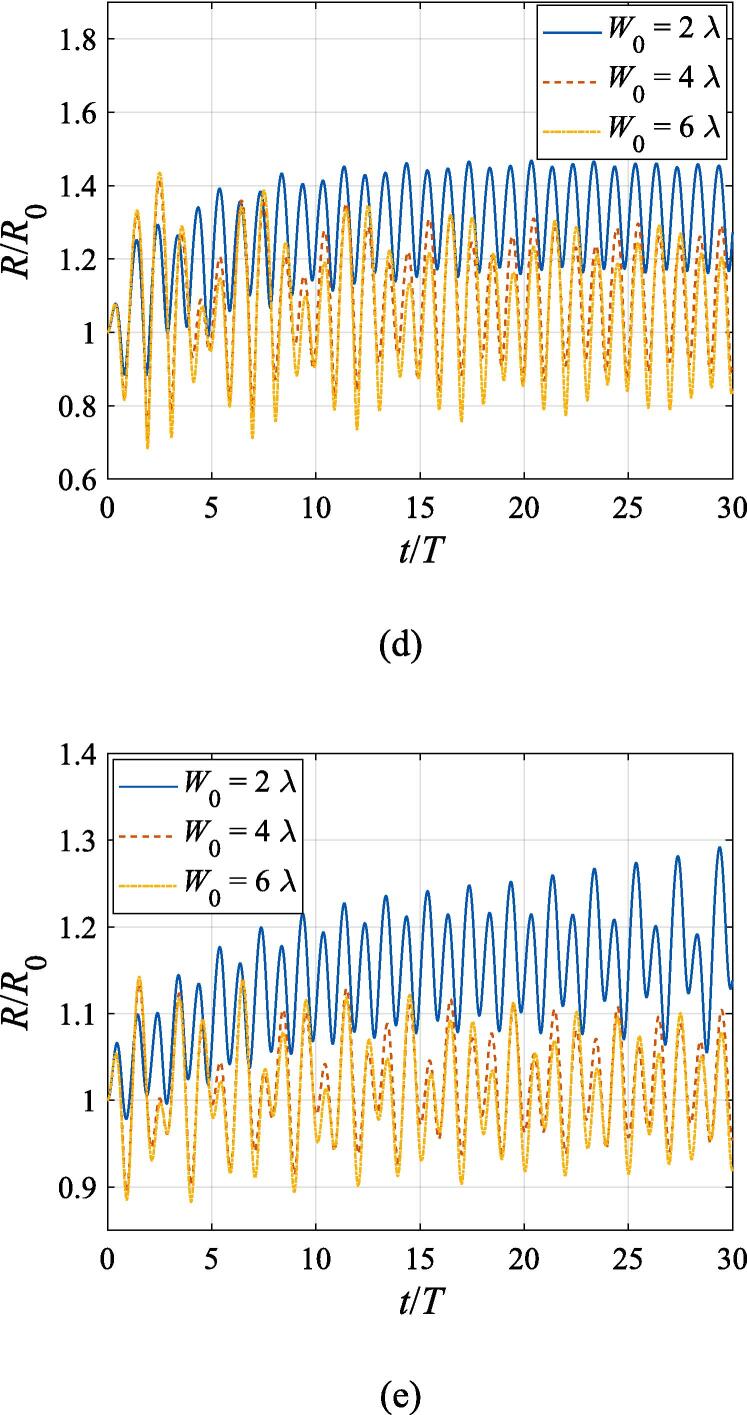
Radial oscillation of a gas bubble in a Gaussian standing wave field for different beam waists with $z_{0}=\lambda /4,x_{0}=\lambda ,A=\text{0.5 bar}$. The driving frequencies in panel (a), (b), (c), (d) and (e) are 137 kHz, 274 kHz, 342 kHz, 411 kHz and 547 kHz, respectively.

**Fig. 11:**
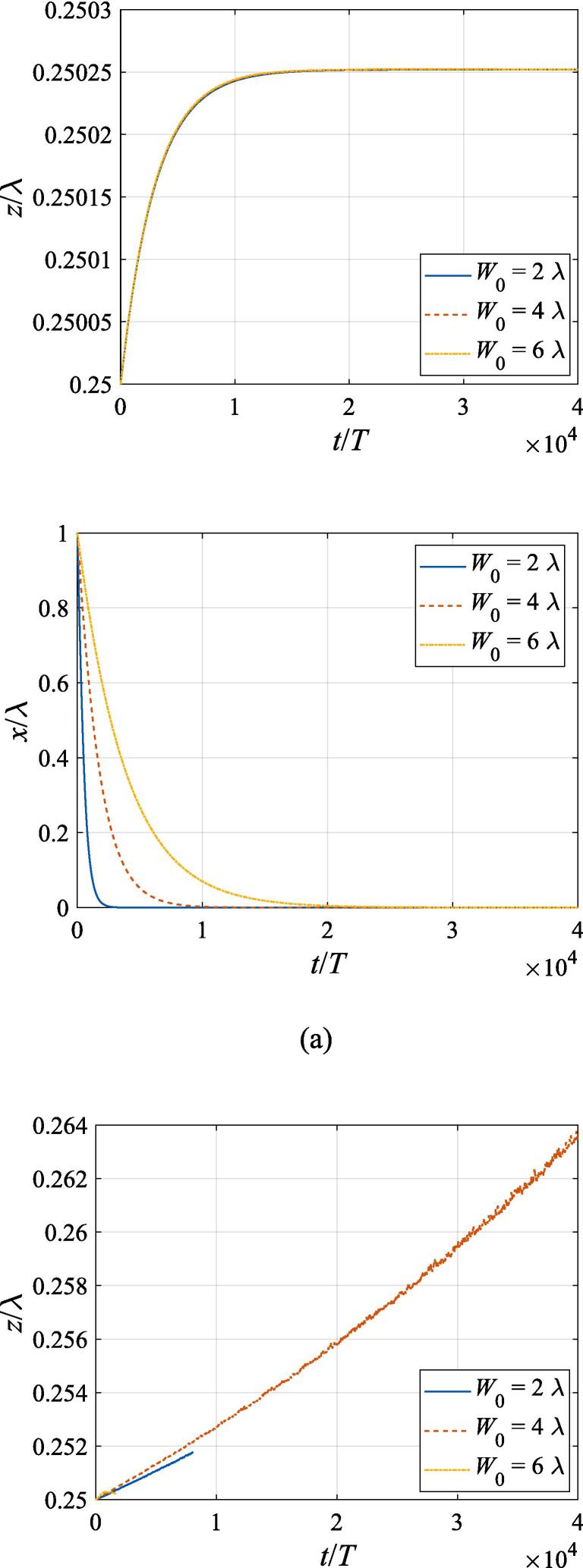

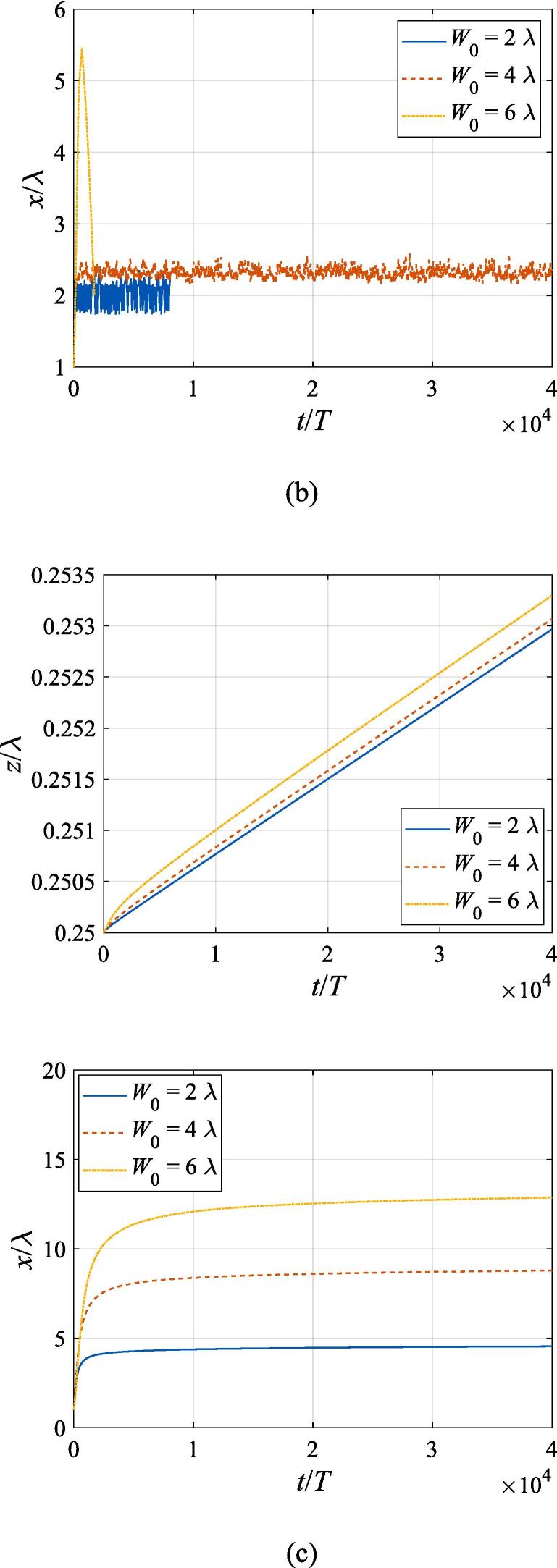

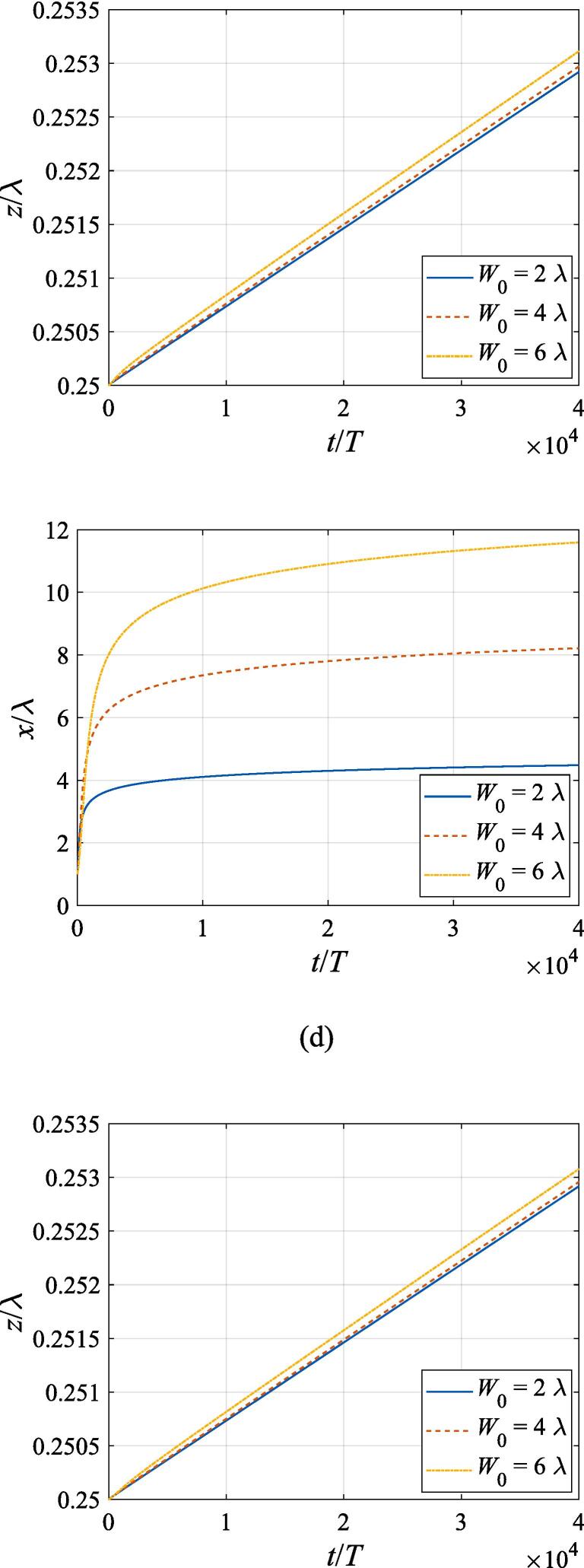

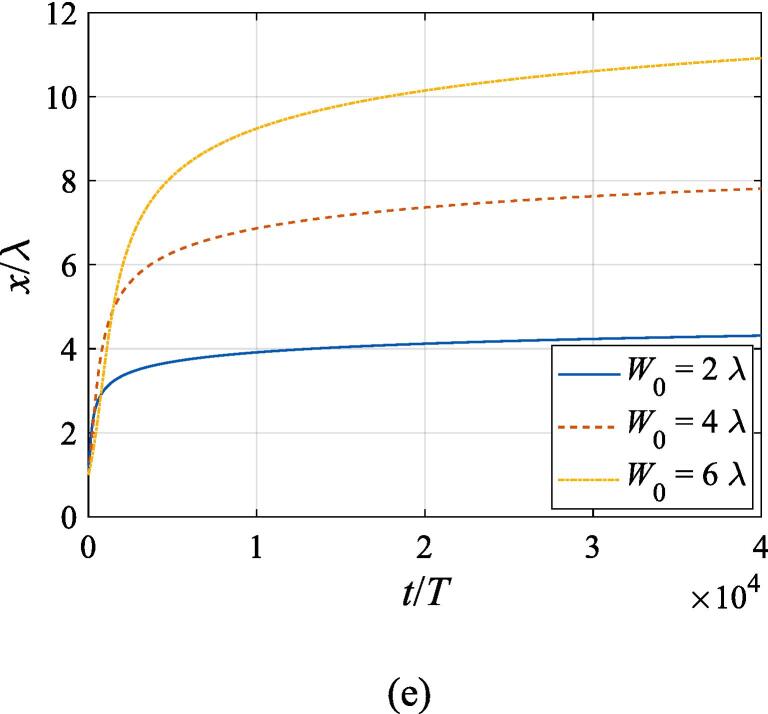
Translational motion of a gas bubble in a Gaussian standing wave field for different beam waists with $z_{0}=\lambda /4,x_{0}=\lambda ,A=0.5\;\text{bar}$. The driving frequencies in panel (a), (b), (c), (d) and (e) are 137 kHz, 274 kHz, 342 kHz, 411 kHz and 547 kHz, respectively.

The effects of the beam waist are studied in Fig. 10, Fig. 11. The radial response of the gas bubble is investigated in Fig. 10 for the cases of $W_{0}=2\lambda ,4\lambda ,6\lambda$, respectively, with the initial coordinate of the bubble set to $z_{0}=\lambda /4,x_{0}=0$ and the pressure amplitude fixed to A=0.5bar. Apparently, the growth of *W*_0_ does not influence the general trend of the simulated plots but leads to an increase in the oscillation amplitude. This is not surprising as the widening of the Gaussian standing wave corresponds a stronger acoustic field around the off-axial sphere and thus the radial response will be intensified. Fig. 11 shows the translational motion at $W_{0}=2\lambda ,4\lambda ,6\lambda$ with the bubble initially put at the same position and the pressure amplitude also fixed to A=0.5bar. For $f=0.4f_{\mathrm{res}}$ (Fig. 11(a)), the widening of the incident wave only affects the transverse motion of the gas bubble. As *W*_0_ increases, a longer time is required for the bubble to be attracted towards the beam axis due to reduction of the pressure gradient. For the same reason, when $f=f_{\mathrm{res}},\;\text{1}.2f_{\mathrm{res}},\;\text{1.6}f_{\mathrm{res}}$ (Fig. 11(c), (d) and (e)), the bubble possesses a smaller transverse velocity for a larger beam waist, whereas it is directed towards the +*x* direction in this case. At $f=0.8f_{\mathrm{res}}$ (Fig. 11(b)), one can observe the irregular oscillation around certain positions though the calculation is terminated again due to divergence. Moreover, the axial motion is hardly affected by changing *W*_0_ irrespective of the driving frequency because the buoyant force and viscous force are the dominant factors for the bubble.

## 4 Conclusions

This study presents a comprehensive formalism on the radial and translational responses of a gas bubble in a Gaussian standing wave field. A coupled system of equations of radial and translational motions are derived. Compared with former studies of the bubble dynamics in plane waves, translational motion in both the axial and transverse directions are required to be taken into consideration as the pressure gradient is nonzero in the transverse plane. Based on the theoretical results, numerical solutions are obtained for radial and translational dynamic behaviors of a gas bubble. The influences of varying the initial position of the bubble and the beam waist of the Gaussian standing wave are also investigated.

Nonlinear radial oscillation is observed in the simulated plots for the gas bubble, which can be intensified by a higher amplitude pressure amplitude and a smaller off-axial distance. For a gas bubble driven much below the resonance frequency, it will reach equilibrium close to the pressure antinode under the action of the buoyant force, viscous drag force and primary Bjerknes force. However, when the driving frequency is not less than the resonance frequency, the bubble will move towards the pressure node under the primary Bjerknes force. Irregular translational oscillation occurs at $f=0.8f_{\mathrm{res}}$, in which case the numerical solution must be terminated ahead of time due to divergence of calculation. In the off-axial configuration, the bubble is pulled towards the beam axis when driven much below the resonance frequency and repulsed away from the beam axis when driven above the resonance frequency. As the beam waist grows, the Gaussian standing wave is widening, which will weaken the radial oscillation of the gas bubble. Besides, a wider wave field induces a smaller pressure gradient and costs the bubble a longer time to reach equilibrium whatever the driving frequency is.

## Declaration of competing interest

The authors declare that they have no known competing financial interests or personal relationships that could have appeared to influence the work reported in this paper.
